# The Current Status of the Use of Internet Hospitals for Outpatients With Pain: Retrospective Study

**DOI:** 10.2196/44759

**Published:** 2023-09-11

**Authors:** Ling Sang, Li Song

**Affiliations:** 1 Department of Pain Management West China Hospital Sichuan University Chengdu China

**Keywords:** internet hospital, internet +, pain management, online visit, outpatient, pain

## Abstract

**Background:**

The national “Internet +” policies and the emergence of internet hospitals have created a new direction for the management of pain outside of the hospital. Nevertheless, there are no consolidated studies conducted by pain physicians on the current state of internet hospital–based online medical services used by patients with pain outside of a hospital setting.

**Objective:**

In this retrospective study, we aimed to examine the status of the use of internet hospitals by patients who experience pain. Moreover, we identified the factors that influenced patients' decisions to make an online visit through the internet hospital.

**Methods:**

Detailed information was collected online and offline from outpatients with pain at the information technology center of West China Hospital of Sichuan University from February 2020 to April 2022. Binary logistic regression analysis was conducted to identify the determinants that influenced patients' decisions to make an online visit to the internet hospital.

**Results:**

Over a 2-year period, 85,266 pain-related clinic visits were recorded. Ultimately, 39,260 patients were enrolled for the analysis, with 12.9% (5088/39,260) having online visits. Both online and offline clinics had a greater number of visits by women than men. The average age of patients attending the online clinic was 46.85 (SD 16.56) years, whereas the average age of patients attending the offline clinic was 51.48 (SD 16.12) years. The majority of online clinic visitors (3059/5088, 60.1%) were employed, and one of the most common occupations was farming (721/5088, 14.2%). In addition, 51.8% (2635/5088) of patients who participated in the online clinics lived outside the hospital vicinity. Young (odds ratio [OR] 1.35, 95% CI 1.01-1.81; *P*=.045) and middle-aged (OR 1.98, 95% CI 1.81-2.16; *P*<.001) patients, employed patients (OR 1.11, 95% CI 1.04-1.18; *P*=.002), nonlocal patients (OR 1.57, 95% CI 1.48-1.67; *P*<.001), and the ordinary staff (OR 1.19, 95%CI 1.01-1.39; *P*=.03) were more likely to have the intention to choose online visits through the internet hospitals.

**Conclusions:**

Internet hospitals are flourishing as a more efficient and promising method of pain management and follow-up for patients with pain outside the hospital. People with pain who are young, working, and not in the vicinity of hospitals are more likely to visit internet hospitals.

## Introduction

### Background

One of the most common reasons for seeking medical care is pain, which can be classified as acute pain or chronic pain. Chronic pain is defined as pain that lasts or recurs for more than 3 months. Over 30% of the world's population experiences chronic pain [1], and in China, 300 million people are living with chronic pain, with a notable prevalence among young individuals [2]. Chronic pain can have a considerable impact on people's lives, causing psychological disorders (eg, anxiety and depression), loss of work capacity, sleep problems, social withdrawal, and economic hardships. Given this, it is economically worth considering chronic pain as a disease. The long-term management of chronic pain remains a challenge, particularly in out-of-hospital settings, despite various treatment options available for pain, as the underlying causative factors are unclear. Due to the demographic and geographic imbalance in the distribution of medical resources in China, there are only 2 doctors for every 1000 people [3]. Therefore, it is impossible to assign every patient a family doctor. In addition, people tend to go to tertiary hospitals, even for minor illnesses, making these hospitals overcrowded and delaying treatment for patients who truly need to go to high-level hospitals. Finally, in the postepidemic era, factors such as avoidance of gatherings, traffic control, and community lockdowns have made out-of-hospital pain management and follow-up an urgent challenge.

With the development of the internet and electronic devices, mobile medicine and telemedicine have emerged. Mobile medical apps for chronic pain management and follow-up are also available on the market. A number of studies [4] suggest that using mobile medical apps for chronic pain management can relieve pain and improve quality of life. However, these apps do not offer features such as disease diagnosis, electronic prescriptions, or checkup appointments nor do they allow physicians or end users to design apps [5]. Therefore, these apps might not be entirely suitable for physicians to use in clinical settings. However, a new health care model that bridges mobile health and telemedicine, known as the internet hospital, has emerged.

### Internet Health Care

Mobile medicine and telemedicine are the main components of internet health care. Internet hospitals currently exist only in China, and there is no such thing as internet hospitals abroad [6,7]. Telemedicine and mobile health are implemented in different forms. Telemedicine refers to the use of telecommunications technology to provide health care services and clinical information, including all medical activities, such as remote diagnosis, remote consultation and nursing, remote education, and remote medical information services. Mobile health refers to the provision of medical services and information through the use of mobile communication technologies, such as PDAs, mobile phones, and satellite communications. The range of mobile health services in the United States is broad, including health management, chronic disease management, and patient health monitoring [8]. Additionally, along with the popularity of electronic medical records, social pharmacies play an important role in the “Internet +” medical care in the United States. Doctors at social pharmacies are able to conduct remote diagnoses and issue prescriptions to residents. The social pharmacy platform allows residents to purchase medications online or pick them up at a nearby pharmacy [9]. According to a questionnaire survey conducted in the United States, patients with long-term follow-ups in pain departments used telemedicine more frequently than patients with no long-term follow-ups, and public hospitals used telemedicine more often than private hospitals [10].

### Internet Hospitals

**Figure 1:**
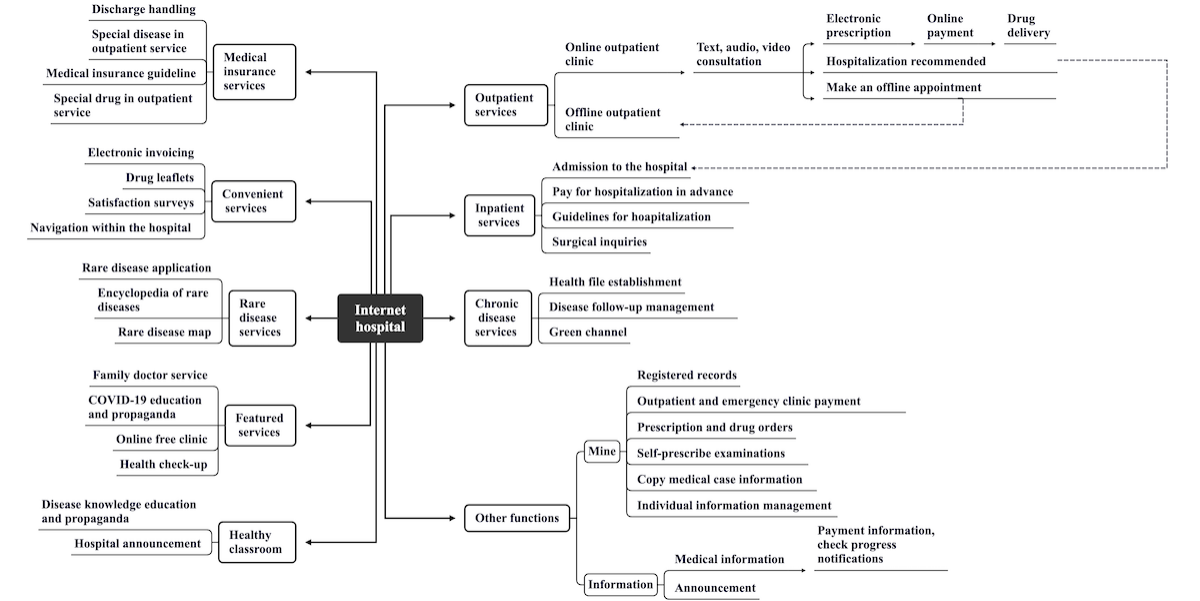
Functions of the internet hospital.

Internet hospitals are a new business model derived from the “Internet +” medical care in China, which is an extension of telemedicine and traditional physical hospitals. Using the medical resources of the physical hospital and internet technology, internet hospitals provide closed-loop online and offline as well as front-end and back-end medical services [11]. In general, the concept of internet hospitals can cover telemedicine and mobile medicine [12,13]. Internet hospitals can serve not only the functions of telemedicine and mobile health but also exercise some functions of physical hospitals. Furthermore, the emphasis on service has some differences; in terms of service recipients, internet hospitals mainly serve patients, while telemedicine can be used both for patients and doctors to achieve remote education and other functions. In terms of service content, internet hospitals can provide patients with all online medical and nonmedical services related to outpatients and hospitalization, while telemedicine mainly provides medical-related services, such as remote diagnoses, remote consultations, and remote education. Currently, in China, patients have internet hospitals at their fingertips. The integration of the internet and medical care began in China with the operation of pharmaceutical e-commerce companies, such as Haodaifu, and the first internet hospital, the Guangzhou Provincial Internet Hospital, was built under the impetus of national policies in October 2014. Although the national “Internet +” implementation policy changed midway, it remained in favor of encouraging the development of “Internet +” [12,13]. In 2018, there were more than 100 internet hospitals in China, and in 2020, the number of internet hospitals increased to 530 as a result of public health emergencies, such as COVID-19. Currently, there are more than 1600 internet hospitals in China [14], primarily affiliated with public hospitals. Internet hospitals are generally divided into 3 types, as follows: enterprise-led, hospital-led, and government-led hospitals, of which public hospital-led internet hospitals are the most popular among Chinese patients [12,15]. West China Hospital of Sichuan University is a representative tertiary hospital in Southwest China. West China Hospital officially adopted West China Hospital Internet Hospital as its second name in October 2019, signifying a hospital-led internet hospital. Using 2 software platforms, the “West China Hospital of Sichuan University” official WeChat account and the “HuaYiTong” app, patients could access professional medical services anytime and anywhere. An internet hospital is a comprehensive platform that can perform most of the functions of a physical hospital, and it helps patients manage their illnesses before, during, and after medical treatments. It offers services like online clinics, appointment registration, intelligent guides, examination appointments, online payments, and drug delivery (Figure 1). By using fragmented time and fixed schedules, doctors can offer online clinic services and perform multidisciplinary diagnosis and treatment for patients with multiple comorbidities, thereby providing authoritative answers and optimal treatment plans to patients. In particular, for patients with chronic pain, internet hospitals can act as a bridge for their long-term pain follow-up and management.

### Study Aims

There have not been any major studies on the use of internet hospitals for out-of-hospital pain management. The aim of this retrospective study was to examine the status of the use of internet hospitals among patients with pain and identify the factors influencing patients’ decisions to seek an online visit through internet hospitals. This study also provides scientific evidence for promoting the use of internet hospitals for pain management.

## Methods

### Data Sources

This is a retrospective study that included all patients who attended the pain Clinic of West China Hospital of Sichuan University between February 2020 and April 2022. The independent variables were the patient's sex, age, working status, occupation, and residence; the dependent variable was whether the patient visited the internet hospital. Both the dependent variables and the independent variable were obtained through the information technology center of West China Hospital of Sichuan University. For analysis, data relating to patients’ age, sex, disease diagnosis, comorbidities, occupation, and place of residence were organized in Microsoft Excel 2016. However, due to incomplete information on disease diagnosis and comorbidities, these 2 variables were not compared between online and offline groups and were not included in the binary logistic regression. The age groups were as follows: younger than 18 years for the underage group, 18 to 44 years of age for the young group, 45 to 64 years of age for the middle-aged group, and 65 years and older for the older group. Regarding employment status, patients in the employed group consisted of those employed by the national civil service, professional and technical staff, general personnel, workers, as well as farmers and freelancers; patients in the unemployed group consisted of those who were not currently employed.

### Statistical Analysis

To analyze the data, IBM SPSS Statistics 27 was used. The mean, SD, and percentages were used to describe the demographic characteristics of the final sample. For differences between online and offline groups, the chi-square test was used for categorical variables and the independent samples 2-tailed *t* test was applied for continuous variables. Binary logistic regression analysis was used to identify the factors that affected patients’ intentions to choose online visits through the internet hospital. Variables with statistical differences between the 2 groups in the bivariate analysis were included in the logistic regression model. The final set of covariables included age, employee status, occupation, and place of residence; the dependent variable was whether or not to visit an internet hospital.

### Ethical Considerations

This study was approved by the ethics committee on the biomedical research of West China Hospital of Sichuan University under the approval number 2022 Review (467); it was also registered on the Chinese Clinical Trial Registry (ChiCTR2200059152). This study did not require informed consent, and the study data were anonymous. There was no compensation provided to the participants.

## Results

### Sample Selection

**Figure 2:**
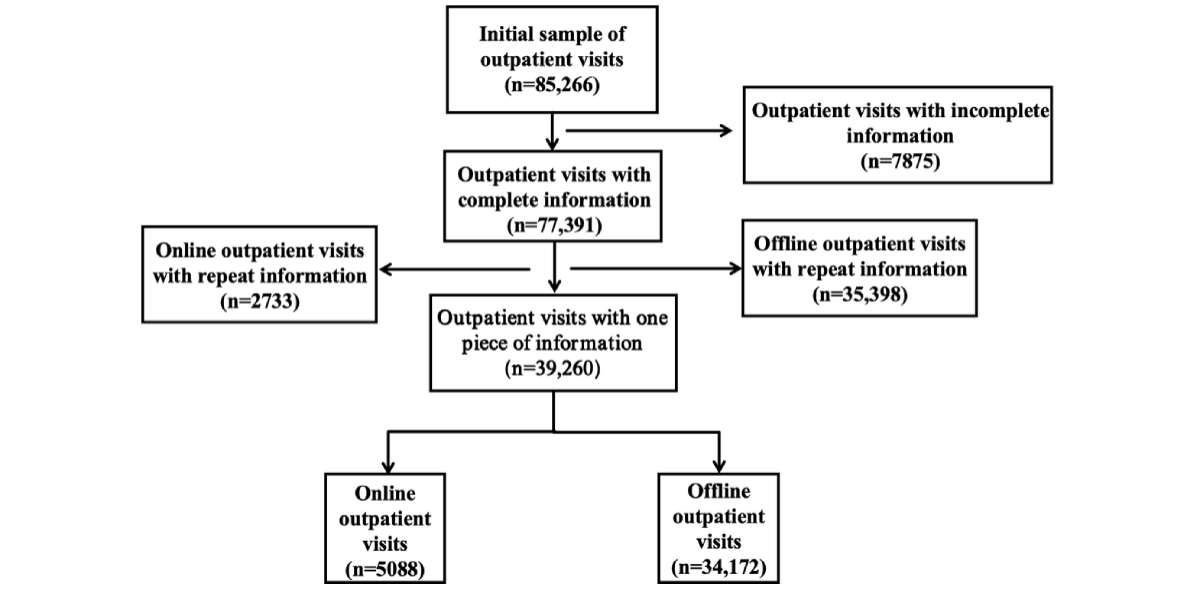
Flowchart of the sample selection.

From February 2020 to April 2022, a total of 85,266 outpatient visits to pain clinics were retrieved. Of these, 7875 outpatient visits with incomplete information were excluded from this study, resulting in 77,391 outpatient visits, including 7821 online visits and 69,570 offline visits. We then screened out repeat visits so that only 1 piece of information was retained for each patient. A total of 39,260 patient records were retrieved, 5088 (12.9%) for the online clinic and 34,172 (87.0%) for the offline clinic (Figure 2).

### Characteristics of Online and Offline Outpatients

From February 2020 to April 2022, a total of 5088 patients with pain opted to conduct online visits at West China Internet Hospital. This represented 12.9% of all pain-related outpatient visits. Table 1 presents the characteristics of outpatients for both online and offline visits. In the pain outpatient clinic, there were more female patients than male patients, but there was no significant difference between online and offline visits. The average age of patients during online visits (46.85, SD 16.56 years) was younger than that of patients during the offline visits (51.48, SD 16.12 years). Young people (2338/5088, 45.9%) were the main population of online visits, while middle-aged people (15,309/34,172, 44.8%) were the main population of offline visits. The majority of patients who visited the pain clinic for help were people who were employed (3059/5088, 60.1%). Among the 9 occupation types, “farmer” was the most common occupation for both online and offline outpatients. There was a significant difference in occupation type between the 2 groups. The majority of patients with pain who visited the online clinic of West China Internet Hospital did not reside in Chengdu (2635/5088, 51.8%), whereas most of the patients who visited the offline clinic resided in Chengdu (19,741/34,172, 57.8%). There was a statistically significant difference in age, working status, occupation type, and place of residence between the online and offline visits. For outpatient disease diagnosis, the most common conditions in online clinics were headache (287/5088, 5.6%), osteoporosis (256/5088, 5.0%), and cancer pain (234/5088, 4.6%), while the most common ones in offline clinics were herpes zoster–related neuralgia (8003/34172, 23.4%), osteoporosis (7152/34,172, 20.9%), and cancer pain (5676/34,172, 16.6%).

**Table 1:** The demographic characteristics of patients in the pain outpatient clinic.

Characteristics		Online visits (n=5088)		Offline visits (n=34,172)	*χ*^2^ or *t* test (df)			*P* value
**Gender, n (%)**						1.758^a^ (1)		.19
	Male	2061 (40.5)	13509 (39.5)					
	Female	3027 (59.5)	20663 (60.5)					
Age (years), mean (SD)		46.85 (16.56)		51.48 (16.12)	19.020^b^ (39258)			<.001
**Age (years), n (%)**						446.505^a^ (3)		<.001
	<18	55 (1.1)	370 (1.1)					
	18-44	2338 (45.9)	10625 (31.1)					
	45-64	1821 (35.8)	15309 (44.8)					
	≥65	874 (17.2)	7868 (23.0)					
**Employed, n (%)**						75.691^a^ (1)		<.001
	Yes	3059 (60.1)	18320 (53.6)					
	No	2029 (39.9)	15852 (46.4)					
**Occupation type, n (%)**						61.384^a^ (8)		<.001
	Civil servant	269 (5.3)	1507 (4.4)					
	Self-employed	213 (4.2)	1189 (3.5)					
	Worker	190 (3.7)	1401 (4.1)					
	Farmer	721 (14.2)	5076 (14.9)					
	Enterprise manager	240 (4.7)	1348 (3.9)					
	Ordinary staff	556 (10.9)	2622 (7.7)					
	Military personnel	8 (0.2)	24 (0.1)					
	Professional and technical staff	517 (10.2)	2870 (8.4)					
	Freelancer	345 (6.8)	2283 (6.7)					
**Local residents, n (%)**						165.260^a^ (1)		<.001
	Yes	2453 (48.2)	19747 (57.8)					
	No	2635 (51.8)	14425 (42.2)					

^a^Chi-square test results.

^b^*t* test results.

### Intention to Choose Online Clinic Visits

The study results indicate that there were differences in age, working status, occupation type, and place of residence between the online and offline visit patients. Therefore, we performed a binary logistic regression analysis to determine the factors that influenced patients to choose online clinics through internet hospitals. Table 2 presents the results of the binary logistic regression analysis. Young (odds ratio [OR] 1.35, 95% CI 1.01-1.81) and middle-aged (OR 1.98, 95% CI 1.81-2.16) people were more likely to make an online visit. Employed patients were more likely to choose online visits than unemployed patients (OR 1.11, 95% CI 1.04-1.18). Workers (OR 0.76, 95% CI 0.62-0.93) and farmers (OR 0.80, 95% CI 0.68-0.93) were less likely to choose online visits, while ordinary staff (OR 1.19, 95% CI 1.01-1.39) were more likely to choose online visits. Place of residence (whether it was in the same city as the physical hospital) was significantly associated with the intention to choose online visits through internet hospitals (OR 1.57, 95% CI 1.48-1.67).

**Table 2:** Binary logistic regression results related to patients’ intention to choose online clinics through internet hospitals.

Characteristics		OR^a^ (95% CI)	*P* value
**Age (years)**			
	<18	Reference	—^b^
	18-44	1.35 (1.01-1.81)	.045
	45-64	1.98 (1.81-2.16)	<.001
	≥65	1.03 (0.94-1.12)	.57
**Employed**			
	No	Reference	—
	Yes	1.11 (1.04-1.18)	.002
**Occupation type**			
	Civil servant	Reference	—
	Self-employed	1.00 (0.83-1.22)	.97
	Worker	0.76 (0.62-0.93)	.007
	Farmer	0.80 (0.68-0.93)	.003
	Enterprise manager	1.00 (0.83-1.21)	.98
	Ordinary staff	1.19 (1.01-1.39)	.03
	Military personnel	1.87 (0.83-4.20)	.13
	Professional and technical staff	1.01 (0.86-1.18)	.91
	Freelancer	0.85 (0.71-1.01)	.06
**Local residents**			
	Yes	Reference	—
	No	1.57 (1.48-1.67)	<.001

^a^OR: odds ratio.

^b^Not applicable.

## Discussion

### Principal Findings

This is a retrospective study that explored the current status of the use of internet hospitals for outpatients with pain in a tertiary hospital and the factors influencing their decision to choose online treatment. According to our study results, 12.9% (5088/39,260) of patients chose to use the internet hospitals for out-of-hospital pain management, and patients who were young, middle-aged, employed, and nonlocal, and those who worked as ordinary staff were more inclined to choose internet hospitals.

In China, patients with pain rarely receive the full range of standardized pain management because they lose contact with their physicians when they leave the hospital setting, compounded by the uneven distribution of medical resources and the impact of COVID-19. There are pain management apps designed for patients experiencing various types of pain, such as cancer pain, low back pain, and post–knee replacement pain [16-18]. However, there are still some problems, such as false advertisements, user information leakage, doctor qualification falsification, medication safety, and user rights protection difficulties [19]. Internet hospitals are targeted at patients with chronic or common diseases [13] and have been successfully applied for glucose management in out-of-hospital patients with diabetes [20]. In addition, internet hospitals can overcome time and geographical restrictions, so it is promising to apply internet hospitals to out-of-hospital pain management and follow-up.

This study, for the first time, examined the current status of the use of internet hospitals by patients with pain. Currently, the number of online clinic visits is much lower than that of offline clinic visits, but internet hospitals are in a phase of rapid and steady growth. The reasons why people prefer to use offline clinics may be that they are currently more accustomed to offline, face-to-face visits and have less trust in online forms of consultation; some people are not even aware of the existence of online clinics. In both online and offline clinics, female patients outnumbered male patients, which is consistent with a higher prevalence of chronic pain among women [1,21]. However, there was no significant difference between the male and female composition of the online visit group or the offline visit group, indicating that sex was not a factor influencing the use of internet hospitals by patients with pain. Although the prevalence of pain is higher among adults older than 65 years compared to the general adult population [22], our study found that young and middle-aged patients dominated online clinic visits. The reasons for these differences were mainly because older patients did not have smartphones or were less receptive to smartphones and mobile apps, coupled with age-related changes, such as vision deterioration. However, in the information age, older patients with pain should not be left behind, and more efforts should be made to help and guide them to use internet hospitals, including improving the internet hospital platform and opening up special areas for aging.

Patients may choose online visits based on their working status and occupation types. In our study, we discovered that patients who were working were more likely to use online clinics. This may be because patients who are working are very busy and using the internet hospital can save them time; on the other hand, people who are working are more likely to be tired and experience pain. At the same time, we found that workers and farmers were less likely than civil servants to choose online outpatient services, while ordinary staff were more likely to choose online outpatient services. This phenomenon might be influenced by differences in education levels, with workers and farmers potentially being less accustomed to smartphone use. However, workers and farmers perform heavy manual labor, and their pain may be greater. In addition, we found that patients with pain who were not in proximity to physical hospitals were more likely to use the online services of internet hospitals, suggesting that internet hospitals can overcome geographical restrictions and be more convenient for patients. In particular, the internet hospital plus drug delivery service can fulfill the medication requirements of patients in remote areas with minor diseases, thereby reducing the pressure of physical hospital visits [3]. In addition, we found that headaches, osteoporosis, and cancer pain were the most common conditions in the online clinic. These conditions are often chronic and can adversely affect the patient's quality of life. Therefore, it is imperative that patients with these conditions seek medical assistance at home as well.

Our results showed that patient age, proximity to physical hospitals, employment status (ie, whether the patients were working), and occupation types influenced patients’ choices to use internet hospitals. Therefore, it is better to initially promote pain management through internet hospitals among young people, employed individuals, and those residing in nonlocal areas and then expand it to other patient groups.

### Advantages of Internet Hospitals

Since the outbreak of COVID-19, there has been a massive boost in the number of internet health care visits, with 60%-95% of out-of-hospital patients reportedly switching from offline to online medical care in countries such as Italy, the United States, and India [7]. The further development of internet hospitals in the postepidemic era is in line with the requirements of the times. Patients seeking treatment in internet hospitals can not only alleviate their anxiety but also avoid the risk of cross-infection caused by crowd gatherings [23]. Simultaneously, this approach reduces the economic burden on patients who would otherwise consult offline hospitals for medical treatment. Most importantly, internet hospitals can overcome time and space limitations and are more economical and convenient for patients to obtain quality medical services in the current situation of uneven distribution of medical resources. Internet hospitals can also provide a pathway for patients with chronic pain to manage the disease standardly outside the hospital.

### Disadvantages of Internet Hospitals

There are several disadvantages associated with patients seeking pain relief through internet hospitals. First, the disease diagnosis made online may be inaccurate because of the inability to perform a physical examination, laboratory tests, and imaging tests during online treatment through internet hospitals [24]. Second, the physical hospital–based internet hospital does not specialize in pain management as a comprehensive platform. Currently, it can only achieve the functions of patient knowledge promotion, online consultations, electronic prescriptions, drug delivery, and appointment registration. The particular needs of patients who experience pain, such as maintaining a pain log and recording pain levels, have not yet been met. Third, electronic prescriptions currently do not include opioids and sedative-hypnotic drugs, and some drugs are not yet covered by health insurance. Fourth, although there is already a special area for older patients on the platform, the acceptance level of older patients is low, and it is still difficult for them to visit the online clinic.

### Prospective

Although the COVID-19 lockdown has now been lifted, it is undeniable that internet hospitals have played an important role in the epidemic in China. Epidemic prevention and control is only one of the objectives of internet hospitals, and in the future, internet hospitals will serve more chronic diseases, such as chronic pain discussed in this study.

In the future, internet hospital and pain follow-up apps can be combined to provide more diversified and comprehensive services to patients with chronic pain on the internet hospital platform. In addition, the establishment of an electronic patient health information database is more conducive for doctors to understand patients’ condition and make a more accurate diagnosis. With the development of artificial intelligence, patients with chronic pain can use wearable devices to evaluate their health status at home [25], gradually overcoming the challenge of being unable to perform physical examinations; moreover, the monitoring data can be connected and integrated with patients’ electronic information to improve patient care.

### Limitations

This study has several limitations. First, we did not collect a great deal of information about the patients, such as their educational level, the length of their illness, or any other factors that may have affected their decision to use an internet hospital. In the collected data, the two possible influencing factors of disease diagnosis and comorbidities were not statistically analyzed due to the irregular and incomplete completion of information by patients. Additionally, this was a single-center study and used only 1 representative tertiary hospital in Southwest China as an example, which may not reflect the situation in other regions. Last, it is still unclear whether internet hospital–based out-of-hospital pain management is effective for patients with chronic pain.

### Conclusions

Currently, out-of-hospital pain management through the internet is becoming increasingly popular, especially in the postepidemic era. Although it is still in the exploration, improvement, and practice stages, additional research is needed to verify its effectiveness in pain management and promote its further development and improvement.

## Abbreviations

OR: odds ratio

## Data Availability

The data sets generated and analyzed in the study are available upon reasonable request from the corresponding author. Private information, such as patient names, hospitalization numbers, and residential addresses will be anonymized.
